# Design and Implementation of a Flexible Chipless RFID Coding Tag Based on Eyeball Structure

**DOI:** 10.3390/s26061903

**Published:** 2026-03-18

**Authors:** Zhen Zhang, Yan Hu, Zhonghui Zhao, Zhuopeng Wang

**Affiliations:** 1 College of Electronic and Information Engineering, Shandong University of Science and Technology, Qingdao 266590, China; zhenzhang0803@163.com (Z.Z.); huyan961225@163.com (Y.H.); wzhuopeng1@sdust.edu.cn (Z.W.); 2 Comprehensive Support Center of the Department of Industry and Information Technology of Inner Mongolia Autonomous Region, Hohhot 010098, China

**Keywords:** chipless RFID tag, bionic, frequency shift coding, circular ring resonance structure, flexible

## Abstract

**Highlights:**

**What are the main findings?**

- A bio-inspired chipless RFID tag based on an eyeball-like multi-ring resonant structure is proposed, enabling joint frequency-shift and graphic coding through branch modulation.
- Within the effective operating bandwidth, the designed flexible tag achieves an encoding capacity exceeding 45 bits while maintaining a compact structure on a polyimide substrate.

**What are the implications of the main findings?**

- The eyeball-inspired hierarchical resonator architecture provides a new design paradigm for high-density chipless RFID encoding under limited spectral resources.
- The demonstrated bending and rotation tolerance indicates suitability for flexible and wearable identification applications where mechanical deformation is unavoidable.

**Abstract:**

In this paper, inspired by the structural characteristics of the human eyeball, a bionically designed circular resonant structure is proposed, and a flexible chipless radio frequency identification (RFID) tag based on this concept is developed. By selectively adding or removing branch structures, the proposed tag achieves controllable resonant frequency shifts and distinguishable geometric pattern variations. Fabricated on a polyimide substrate with a compact size of 20 × 26 × 0.2 mm^3^, the tag achieves a coding capacity exceeding 45 bits while operating within an effective frequency bandwidth in 4–12 GHz, realizing a synergistic improvement in coding capacity and structural compactness under limited spectrum constraints. Simulation analyses are performed to investigate the encoding stability of the tag under various bending and rotational conditions relevant to flexible applications. Experimental results obtained under the unbent condition are consistent with the simulations, demonstrating the feasibility of the proposed chipless RFID tag.

## 1. Introduction

Chipless radio frequency identification (RFID) technology has attracted increasing attention due to its low fabrication cost, elimination of silicon chips, and suitability for large-scale deployment. Compared with conventional chipped RFID systems, chipless RFID tags generally offer longer operational lifetimes and better adaptability to flexible substrates and harsh environments [1,2]. However, because information encoding relies on passive resonant structures, the coding capacity, physical size, and spectral occupation of chipless RFID tags are often mutually constrained, posing challenges for high-density encoding under limited spectral resources [3,4,5]. With the rapid development of wireless communication technologies and the increasing scarcity of available frequency bands, achieving compact tag size while maintaining high coding capacity has become a key issue in chipless RFID tag design.

To address these challenges, extensive research has been conducted on encoding methods for chipless RFID tags. In chipless tag design, tags with special graphic features are usually encoded based on frequency. The structural parameter value of the tag corresponds to a unique resonant frequency to achieve specific coding. A tag designed with Latin alphabet characters proposed in paper [6] can achieve unique identification with high accuracy. In paper [7], multiple QR code resonators were printed on FR4 substrate on a single side, and coded information was retrieved and identified using a frequency-domain reflection method. Paper [8] realizes frequency shift keying coding through small metal units, proposes chipless tags based on genetic algorithm optimization technology, producing a series of RFID tags with QR code appearance. In paper [9], the open-loop resonator with fragment-loading structure was applied to the design of chipless tags for the first time, which was coded by the distribution of fragment patches in the open-loop resonator. Paper [10] proposed a design scheme for embedding chipless tags in QR codes, where the resonant frequency can be adjusted to encode by removing or loading the square modules.

Mixed coding is one of the most effective methods to improve coding capacity at present. In paper [11], a mixed-coding tag is designed by using a microstrip spiral multi-resonator and a cross-polarized microstrip ultra-wideband disk monopole antenna. In paper [12], a U-shaped resonance structure was applied to achieve 22.9-bit coding capacity on a 2 × 4 cm^2^ FR4 substrate. In paper [13], the tag with an 8-bit coding capacity was designed by using the open-circuit stub connected to the microstrip transmission line. Tags have a compact structure and are encoded in a mixture of amplitude and delay. In paper [14], several concentric nested square rings were used to design and realize the tag with 10-bit encoding capacity. Paper [15] designed a tag printed on a 48 × 48 mm^2^ PET substrate with five improved complementary open-loop resonators, achieving a 19-bit coding capacity.

To overcome the limitations of conventional regular geometric structures in chipless RFID tag design, bio-inspired design concepts have attracted increasing attention in recent years [16,17]. Previous studies have shown that bio-inspired structural modeling approaches have been widely applied in antenna design, achieving favorable results in terms of size reduction, bandwidth enhancement, and electromagnetic performance optimization [18,19]. Therefore, by abstracting and reconstructing natural biological structures, extending bio-inspired structural modeling to chipless RFID tag design can help expand design flexibility and provide new insights into improving encoding performance under limited spectral resources.

Based on this research background, biological structures with multifunctional and hierarchical characteristics offer a feasible reference for the design of high-capacity chipless RFID tags. In this paper, a flexible chipless RFID coding tag inspired by the human eyeball is proposed. By combining graphic coding and frequency-shift coding, the presence or absence of branches is utilized to control resonant frequency variations and pattern changes. As a result, the proposed design reduces spectral occupation while increasing encoding capacity, achieving 45 bits within 4–12 GHz.

## 2. Resonance Structure Design and Analysis

**Figure 1:**
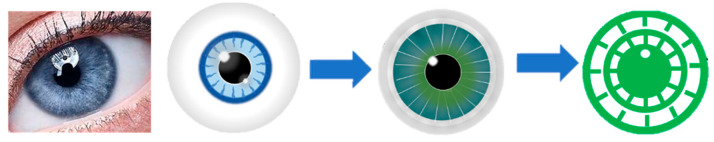
Pictograph and simplification of the eyeball structure [20].

In this paper, a chipless RFID tag based on an eyeball structure is proposed based on bionic thought. As shown in Figure 1, the eye anatomy is pictorially abstracted and simplified [19] to form three resonant sub-structures. Structures I and II are cornea–pupil-inspired ring resonators that encode information by adding or removing radial branches to generate distinct frequency shifts and patterns. Structure III is an eyelid-orbit-inspired orbital resonator that provides an additional tunable resonance to enlarge the coding space. These three parts constitute a hierarchical hybrid coding architecture for tag identification.

**Figure 2:**
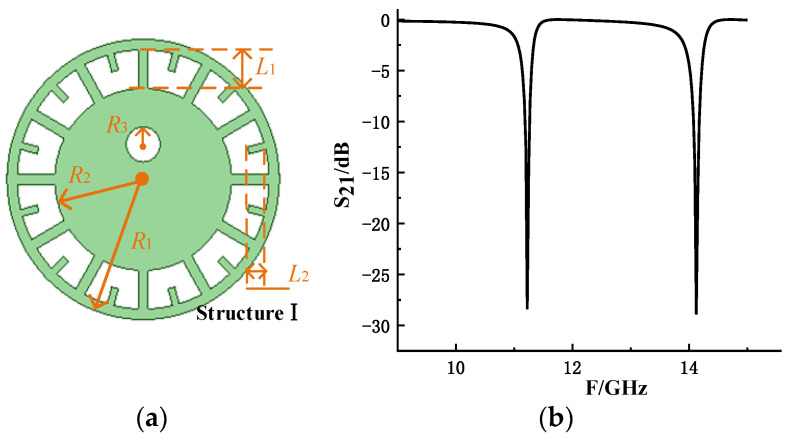
Resonance structure I based on the eyeball: (**a**) structure chart; (**b**) S_21_ parameters.

First, Resonant Structure I is designed by mimicking the cornea–pupil region and its surrounding area, as shown in Figure 1. The white circular slot in the middle is used to simulate the pupil, and it has little effect on the resonant frequency of the resonant structure. Its main function is to mark the direction of the resonant structure. Figure 2 shows S_21_ of the resonant structure. The resonant frequency is affected by the branch length *L*_2_ and the ring radius *R*_1_. The structure is a double resonant structure, but only one resonant frequency point is in the frequency band range required by the design, and the two resonant frequency points have similar changes with the structure, so only the first resonant frequency point is used for coding.

**Figure 3:**
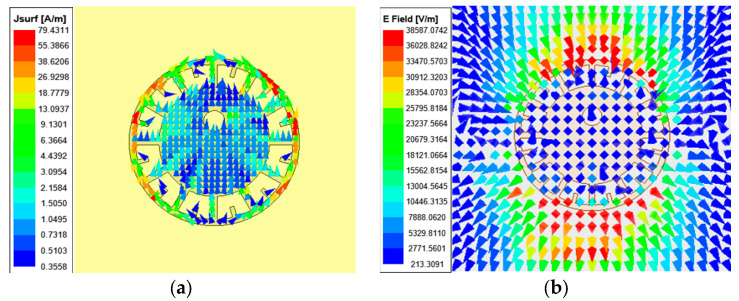
Current distribution and electric field distribution of resonance structure I at 11 GHz: (**a**) current distribution; (**b**) electric field distribution.

Figure 3 shows the current distribution and electric field distribution of the resonant structure at 11 GHz. The current distribution in the outer ring of the resonant structure is the same as that in the traditional ring resonant structure. And the current converges from the outer ring to the inner ring, and the intensity of the inner current is obviously lower than that of the outer ring. According to the electric field distribution diagram, the outer ring electric field of the eyeball resonance structure I is strong, and the intensity is strongest at the upper and lower vertices of the ring.

**Figure 4:**
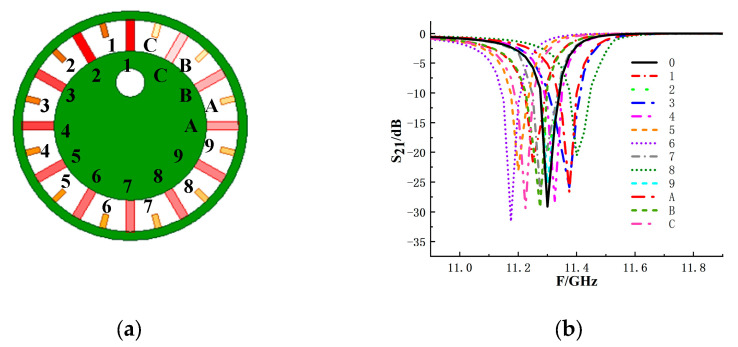
Resonant frequency characteristics of the eyeball resonance structure I with different branches removed: (**a**) schematic diagram of the structure I with branch numbering; (**b**) simulated S_21_ responses corresponding to the removal of branches.

The eyeball resonance structure I contains multiple radial-shaped branches. When excited, each branch generates a different induced current and electric field, so different resonant frequencies are obtained when different branches are removed or added. Each branch is unique and clearly identifiable. Therefore, tag encoding can be realized by both a frequency domain and a graph. The combination of the two methods can effectively improve the reliability of tag reading. Figure 4 shows the resonant frequencies corresponding to removing branches 1~12. In fact, the eyeball resonance structure I has 24 branches. According to the simulation results, it can be seen that some branches, such as “1” and “3”, have the same resonant frequency, so it is necessary to remove these repeated branches in coding.

**Figure 5:**
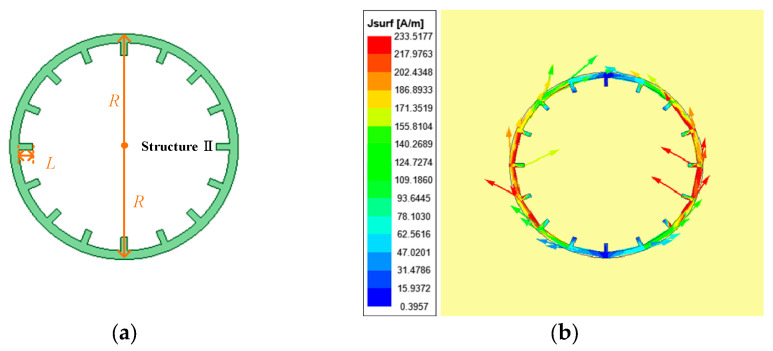
Resonance structure II based on the eyeball: (**a**) structure chart; (**b**) current distribution.

**Figure 6:**
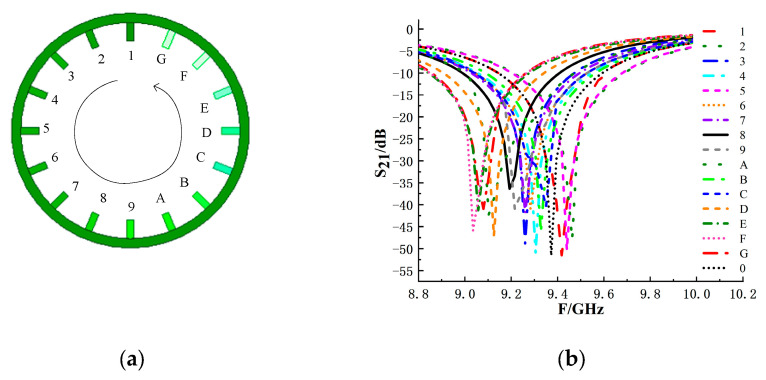
Resonant frequency responses of eyeball resonance structure II with individual branches removed: (**a**) structural schematic of the structure II with branch numbering; (**b**) simulated S_21_ responses corresponding to frequency shifts.

To further extend the encoding capability, resonant structure II is designed, as shown in Figure 5a. Similar to the coding method of eyeball resonance structure I, the coding method of eyeball resonance structure II also adopts the addition and removal of branches. According to the current distribution shown in Figure 5b, it can be seen that resonant structure II and resonant structure I have similar current characteristics. The current distribution is significantly affected by the position of the feeding point and the ratio of the ring circumference to the wavelength. The current reaches its maximum value at the feeding point, while at 90°/270°, the current is at its minimum. This distribution indicates that the current intensity is a function of angle, reaching its maximum near the feeding point and monotonically decreasing to its minimum as the angle changes. Therefore, adding branches within 360° of the inner circle of the circular structure can adjust the resonance situation. The resonance frequencies corresponding to the tags after removing different branches are analyzed by simulation, as shown in Figure 6. Starting from the branches at the highest point of the ring, the code is successively coded in the counterclockwise direction 1~16. It can be seen from the simulation results that each branch corresponds to a different resonant frequency, that is, corresponding to a different frequency shift size.

**Figure 7:**
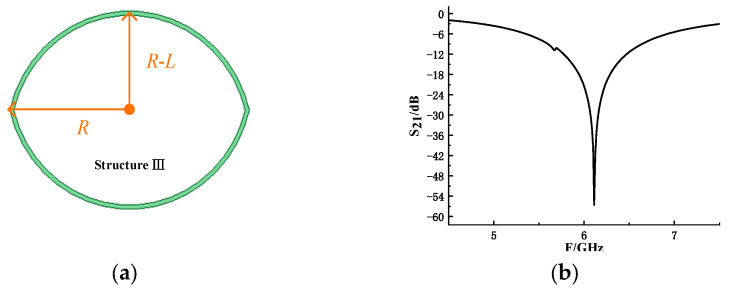
Resonance structure III based on orbital: (**a**) structural schematic of the structure III; (**b**) simulated S_21_.

In addition to the ring-based resonant structures, an orbital-shaped resonant structure is introduced to emulate the eyelid region of the eye, as shown in Figure 7a. The structure is the most external of the whole design, and the available physical area is larger, so the coding by changing the radius to change the resonant frequency is less limited.

**Figure 8:**
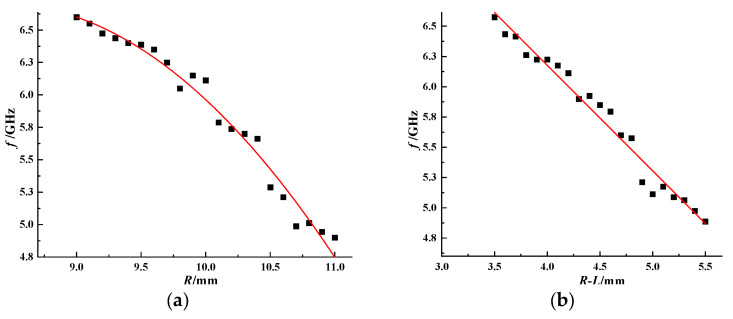
Resonant frequency of the orbit-shaped resonant structure as a function of (**a**) radius *R*; (**b**) opening *R*-*L.*

The simulation model is established in electromagnetic simulation software, and the values of radius and resonance structure are changed to obtain the result shown in Figure 8. The resonant frequency of the resonant structure changes regularly with *R* and *R*-*L*. The relationship between them can be described by Equations (1) and (2):

(1) $$f=-A_{1}\times R^{2}+A_{2}\times R-A_{3}\left(9\leq R\leq 11\right)$$

where *A*_1_ = 0.29, *A*_2_ = 4.865, *A*_3_ = 13.73, *R*^2^ = 0.97443 (Unit: mm).

(2) $$f=-B_{1}\times {\left(R-L\right)}^{2}+B_{2}\left(3.5\leq R-L\leq 5.5\right)$$

where *B*_1_ = 0.872, *B*_2_ = 9.664, *R*^2^ = 0.971 (Unit: mm).

Specifically, as *R* increases from 9 mm to 11 mm, the resonant frequency decreases following a quadratic trend described by Equation (1). Similarly, as the opening *R*-*L* increases from 3.5 mm to 5.5 mm, the resonant frequency also decreases according to Equation (2). These results indicate that both the overall size and the effective gap of the resonant unit can be used to precisely tune the resonant frequency. Therefore, adjusting *R* and *R*-*L* provides a reliable method to design coding tags with desired frequency characteristics.

## 3. Code Tag Design

### 3.1. Encoding Tag Structure

**Figure 9:**
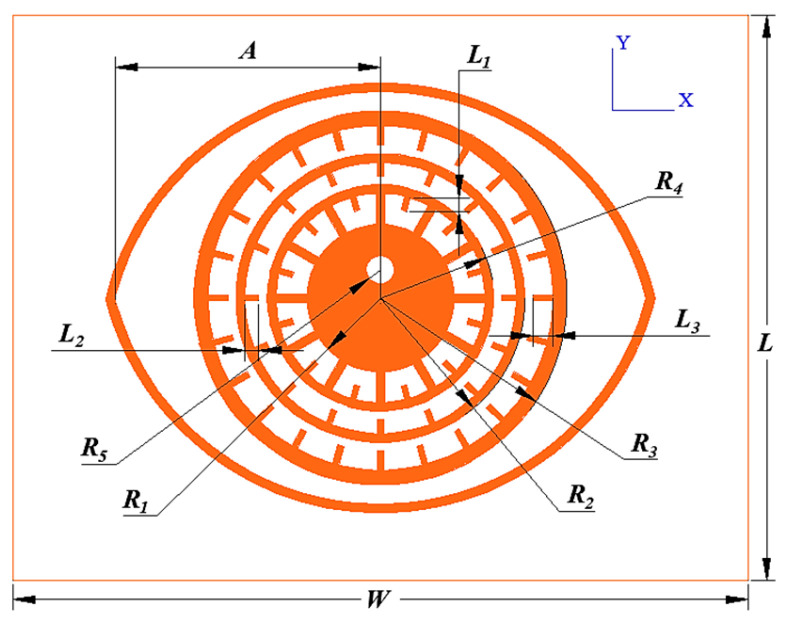
Chipless RFID tag based on the eyeball structure with four concentric resonant layers (substrate thickness *H* = 0.2 mm, size 20 × 26 mm^2^).

By integrating the previously proposed bio-inspired resonant structures, the circular patch is changed into rings with different radii nested together to construct coding tags. The structure of the tag is shown in Figure 9, and the main structural parameters and dimensions of the tag are shown in Table 1. It is composed of four layers of resonant units in concentric nesting mode. The tag uses 0.2 mm-thick polyimide material with a relative permittivity of 3.1 and a loss tangent of 0.002 as the substrate, and its overall size is 20 × 26 mm^2^.

### 3.2. Analysis of Coding Performance of the Tag in the Bending State

**Figure 10:**
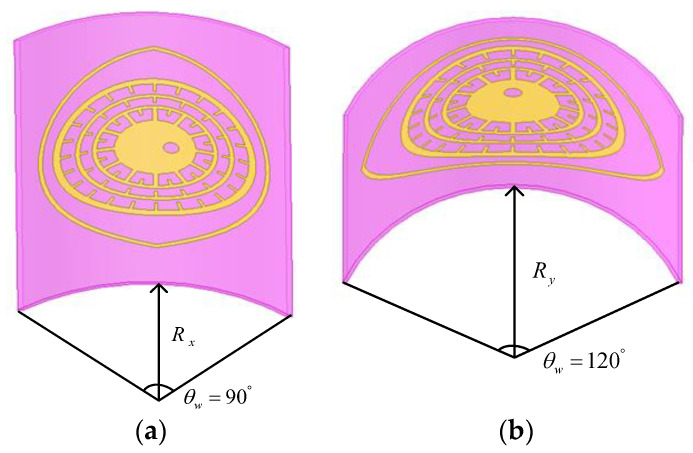
Schematic diagram of the bending state of the coding tag: (**a**) the tag is bent along the X-axis; (**b**) the tag is bent along the Y-axis.

**Figure 11:**
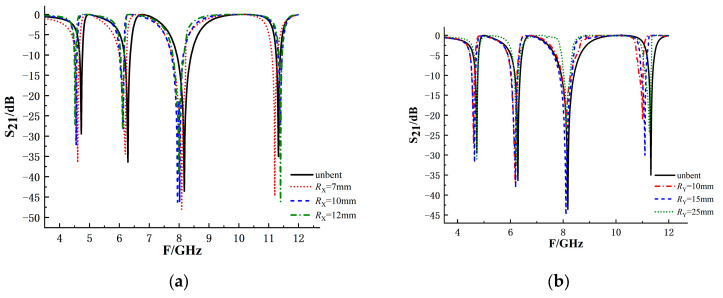
Simulated S_21_ responses of the coding tag under different bending conditions: (**a**) tag bent along the X-axis; (**b**) tag bent along the Y-axis.

In order to study the reliability of the flexible chipless coded tag designed in this paper after bending by force, the influence of different bending degrees on the performance of the tag is simulated and analyzed. As shown in Figure 10, bend the tag along the X and Y axes to different degrees. Where Rx and Ry are the distances between the center of the tag side and the origin of the coordinate when the tag is bent along the X and Y axes. Bend the tag in different degrees to obtain S_21_, as shown in Figure 11.

According to the simulation results, when the tag is bent along the X-axis, the offset degree of the four resonant frequency points increases with the increase in the bending angle. Comparing the four resonant troughs, the frequency offset of the first resonant frequency points after bending is the largest, and the fourth one is the smallest. This is because during the bending process, the outer ring resonant structure is subjected to the maximum bending degree, while the inner ring structure is subjected to the minimum bending degree. When the tag is bent along the Y-axis, the offset degree of the resonant frequency points of the inner ring resonance structure is larger than that of the tag bent along the X-axis. The offset degree of the resonant trough of the outer ring resonant structure is smaller than that of the X-axis bending. Although different bending directions affect the resonant structures at different layers, the resonant frequency points remain distinguishable.

### 3.3. Analysis of Coding Performance in Different Polarization Directions

**Figure 12:**
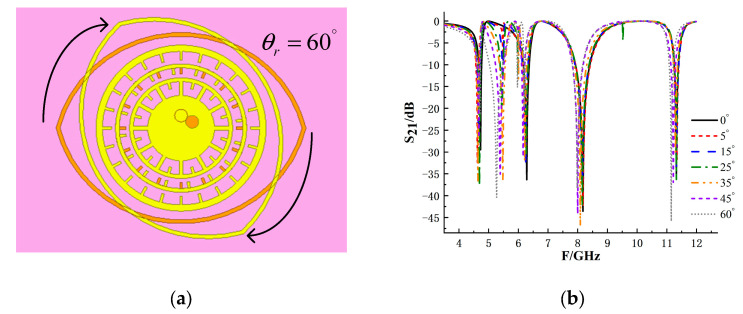
Effect of rotation on the coding tag performance: (**a**) schematic diagram of the tag rotated 60° clockwise; (**b**) simulated S_21_ response under different rotated conditions.

In order to verify the influence of different polarization directions on tag encoding performance, the S_21_ of tags at different rotation angles were simulated and compared. Rotate the tag 60° clockwise as shown in Figure 12.

According to the simulation results, when the tag rotates in the clockwise direction of 0°~35°, the four resonant points almost have no deviation. When the rotation continues to 60°, the tag generates more interference frequency points, and the offset of each frequency point increases to more than 100 MHz, resulting in the reading error of the encoded tag. In the case of counterclockwise rotation, the overall variation in S_21_ with rotation angle is similar to that in the case of clockwise rotation, but the interference resonance trough will occur earlier. These results indicate that, within a moderate rotation range, the resonant frequency points remain distinguishable, which is sufficient for identifying the encoded information.

## 4. Encoding Principle of the Tag

The tag realizes both frequency shift and image change through the presence or absence of each branch. In this way, the accuracy of tag reading in the line-of-sight range is double guaranteed. It can not only read the frequency of the tag by way of backscattering, but also judge the coding information by image recognition.

**Figure 13:**
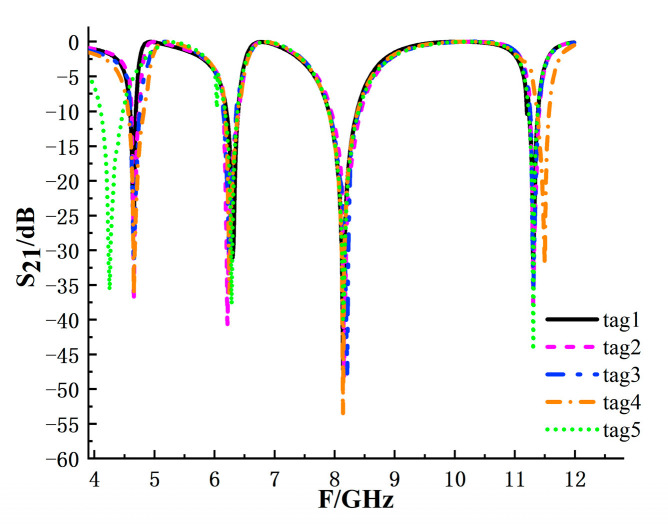
Simulated S_21_ responses corresponding to five groups of coding tags.

To maintain a consistent interrogation band, the operating range used for coding is set to 4–12 GHz. In decoding, each code state is identified by extracting the notch frequencies from the S_21_ response. Two code states are regarded as distinguishable when their resonance frequencies are separated by at least 100 MHz, and the notch depth satisfies a predefined detection criterion under noise. For the overall coding capacity, Structures I and II adopt branch-based binary encoding; after excluding duplicated branches that yield indistinguishable responses, Structure I retains 12 effective branches and Structure II provides 16 branch positions, resulting in 44 bits from Structures I and II. Structure III provides two resolvable geometric states within 4.0–5.5 GHz, contributing 1 bit. Therefore, the overall coding capacity reaches 45 bits, which is theoretical and simulation-based in this work. Figure 13 shows the simulated responses of five representative tags, and the corresponding resonance frequencies used for decoding are summarized in Table 2.

## 5. Measurement

**Figure 14:**
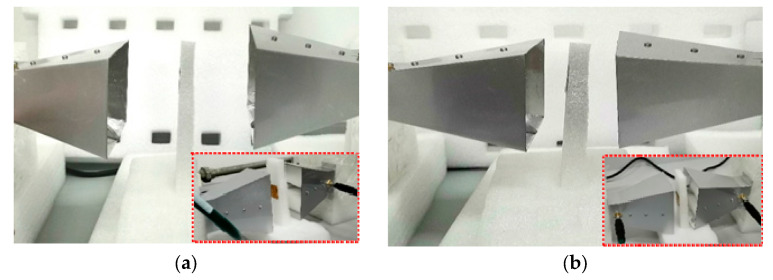
Measurement environment: (**a**) the distance is 16 cm; (**b**) the distance is 8 cm.

As shown in Figure 14, the network analyzer is used as the reader of the tag, and the horn antenna with a bandwidth of 2~12 GHz is used as the transmitting and receiving antenna, connected to the two ports of the vector network analyzer (Keysight E5063A) to build the S_21_ parameter test platform.

**Figure 15:**
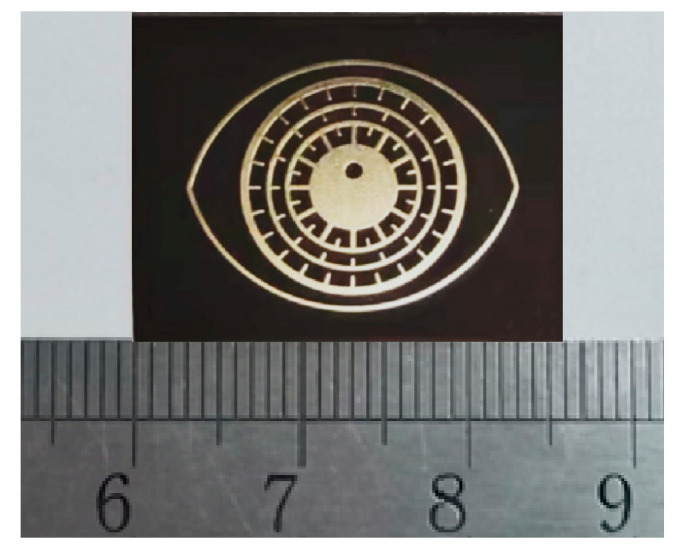
Photograph of a chipless coded tag.

**Figure 16:**
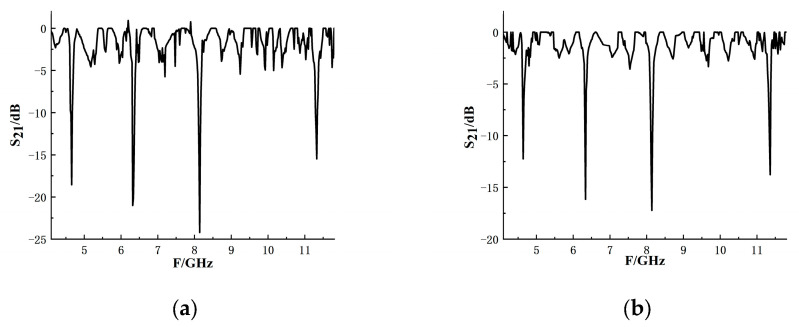
Measurement results after background removal: (**a**) the distance is 8 cm; (**b**) the distance is 16 cm.

Figure 15 is a physical view of the tag. Figure 16 shows the measured results after eliminating the test background. The results show that when the test distance is close, the resonant frequency amplitude of S_21_ is higher, but the amplitude of the interference background is also higher. When the reading distance becomes longer, the amplitude of the S_21_ interference background obtained is small, but the amplitude of the resonant frequency also becomes smaller, and the frequency shift becomes larger.

**Figure 17:**
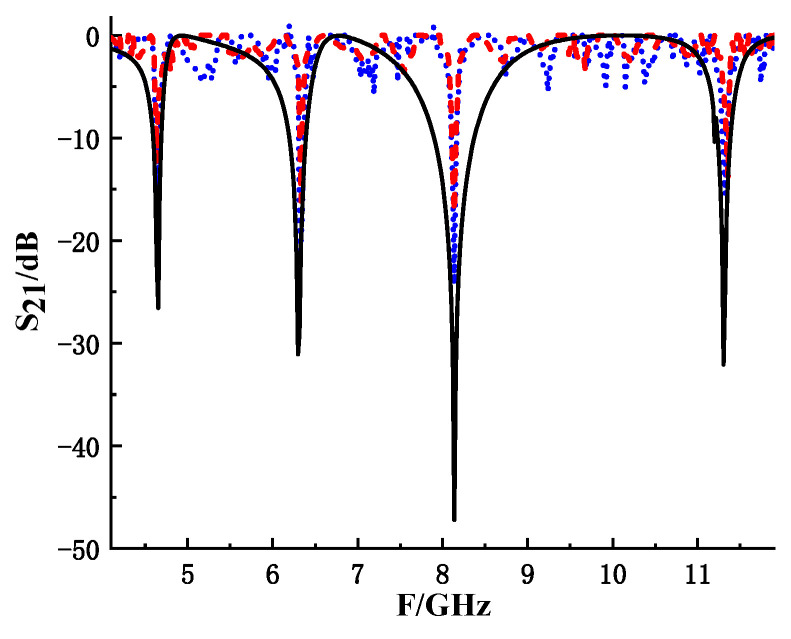
Comparison between tag simulation results and measurement results.

The simulation results of the labels were compared with the measurement results, as shown in Figure 16 and Figure 17. It can be seen that the measurement and simulation are basically consistent. When the testing distance is 16 cm, the offset of the high-frequency resonance point (10–12 GHz) reaches 45 MHz. The frequency stability of the 12 GHz reading antenna is relatively poor, resulting in differences between the measured results and simulation results. Therefore, in the test, the distance between the antenna and the tag will be controlled within 16 cm, which is sufficient for most application scenarios. Meanwhile, due to the fact that the actual testing environment is not an ideal space, the signal experiences greater attenuation during the radiation process, resulting in a decrease in amplitude. So, compared with the simulation results, the S_21_ amplitude obtained from the test is smaller than the simulation data. In addition, due to limited technological capabilities, there may be a difference of ±10% in the accuracy of label production, which can also lead to insufficient resonance amplitude loss and frequency deviation.

A comparison with previously reported chipless RFID tags is summarized in Table 3. Compared with the designs in [21,22,23,24], the proposed tag achieves a higher encoding capacity while maintaining a compact physical size. In paper [21], three circular structures are combined to design a clover-shaped resonant structure, and the designed tag can achieve 10-bit coding capacity on RT/Duroid5880 substrate. The tag frequency range proposed in paper [22] is the smallest, but the deficiency is that the tag size is large. In contrast, the proposed tag adopts a circular ring resonant structure with a compact size of 2.0 × 2.6 cm^2^, while providing an encoding capacity exceeding 45 bits.

## 6. Conclusions

This paper proposes a chipless RFID tag inspired by the eyeball structure based on a bionic design concept. By abstracting and simplifying the structural characteristics of the pupil, iris, and eyelid of the eyeball, resonant units composed of ring structures and radial branches are constructed, enabling a dual-domain encoding mechanism that combines frequency-shift coding and image-based coding through the addition or removal of branches. The proposed tag at 4~12 GHz with an encoding capacity exceeding 45 bits. An encoding density of 8.654 bit/cm^2^ was achieved, reflecting its performance in terms of area utilization and spectral efficiency. Simulation and experimental results are generally consistent, validating the feasibility of the proposed design.

## Figures and Tables

**Table 1:** Structural parameters of the eyeball-based coding tag.

Parametric	*L*	*W*	*H*	*R*_1_	*R*_2_	*R*_3_
/mm	20	26	0.2	3	4	5.3
Parametric	*R*_4_	*R*_5_	*L*_1_	*L*_2_	*L*_3_	*A*
/mm	6.8	0.5	0.8	0.8	1.2	10

**Table 2:** Resonant frequencies corresponding to five groups of tags.

Tag	Resonant Frequency/GHz			
Tag 1	4.655	6.3	8.1375	11.305
Tag 2	4.655	6.2125	8.155	11.305
Tag 3	4.655	6.2475	8.2075	11.3225
Tag 4	4.778	6.318	8.1375	11.4975
Tag 5	4.69	6.2825	8.1375	11.305

**Table 3:** Comparison of different types of chipless RFID tags.

Ref.	Structure	Band	Size (cm^2^)	Substrate	Capacity
[21]	Square open ring	1.5~6.7 GHz	2.4 × 2.4	FR4	3 bits
[22]	Clover-shaped slot	5.4~10.4 GHz	1.35 × 1.35	RT/duroid5880	10 bits
[23]	Circular ring slot	2.0~4.0 GHz	4.0 × 4.0	RO4003	6.97 bits
[24]	Circular ring	3.1~10.6 GHz	3.0 × 3.0	RO4003	19 bits
[15]	MCSRR	0.9~2.7 GHz	4.8 × 4.8	PET	19 bits
This Work	Circular ring	Piecewise	2.0 × 2.6	PI	45 bits

## Data Availability

The data used to support the findings of this study are available from the corresponding author upon request.
